# Supplementary material for: Exploring the effects of degraded vision on sensorimotor performance
Source: PLoS One. 2021 Nov 8;16(11):e0258678. doi: 10.1371/journal.pone.0258678 (PMC8575268; doi:10.1371/journal.pone.0258678)
Supplement: S1 Table — Showing participants with normal VA (Normal); participants with high visual acuity (VA > 0.2 logMAR in with eye; High); Those with a large interocular difference (a difference in VA > 0.2 logMAR between their eyes; Different). WG–With Guide, NG–No Guide, RMSE–Root Mean Squared Error. (DOCX) [file pone.0258678.s001.docx]

| Mean (sd)  [min, max] | Normal  (n = 55) | High  (VA > 0.2 either eye) (n = 11) | Different (VA diff > 0.2 between eyes) (n = 11) |
| --- | --- | --- | --- |
| Tracking NG (RMSE)  - Worse Eye | 9.76 (3.64)  [5.60, 28.35] | 8.94 (1.57)  [6.71, 12.05] | 8.93 (1.39)  [7.14, 11.72] |
| Tracking NG (RMSE)  - Better Eye | 9.52 (2.79)  [5.89, 20.20] | 8.99 (2.31)  [6.24, 14.00] | 8.72 (2.18)  [6.58, 14.00] |
| Tracking NG (RMSE)  - Both Eyes | 9.25 (3.58)  [5.86, 27.91] | 7.81 (1.38)  [5.94, 10.71] | 8.33 (2.01)  [5.92, 12.15] |
| Tracking WG (RMSE)  - Worse Eye | 11.29 (9.42)  [4.84, 56.59] | 7.35 (1.60)  [5.25, 10.76] | 7.53 (1.67)  [5.42, 10.99] |
| Tracking WG (RMSE)  - Better Eye | 10.09 (5.57)  [4.81, 35.79] | 7.43 (1.74)  [5.00, 10.48] | 7.36 (1.62)  [5.00, 10.39] |
| Tracking WG (RMSE)  - Both Eyes | 10.23 (6.51)  [4.99, 38.67] | 7.01 (1.28)  [4.95, 9.23] | 8.87 (4.19)  [4.95, 20.82] |

***S1 Table.* Grouped means (sd) [min,max] for Tracking data.** Showing participants with normal VA (Normal); participants with high visual acuity (VA > 0.2 logMAR in with eye; High); Those with a large interocular difference (a difference in VA > 0.2 logMAR between their eyes; Different). WG – With Guide, NG – No Guide, RMSE – Root Mean Squared Error.
