# Supplementary material for: Exploring the effects of degraded vision on sensorimotor performance
Source: PLoS One. 2021 Nov 8;16(11):e0258678. doi: 10.1371/journal.pone.0258678 (PMC8575268; doi:10.1371/journal.pone.0258678)
Supplement: S2 Table — Showing participants with normal VA (Normal); participants with high visual acuity (VA > 0.2 logMAR in with eye; High); Those with a large interocular difference (a difference in VA > 0.2 logMAR between their eyes; Different). MT–Movement Time (s). (DOCX) [file pone.0258678.s002.docx]

| Mean (sd)  [min, max] | Normal  (n = 55) | High  (VA > 0.2 either eye) (n = 11) | Different (VA diff > 0.2 between eyes) (n = 11) |
| --- | --- | --- | --- |
| Aiming (MT)  - Worse Eye | 1.14 (0.15)  [0.85, 1.57] | 1.09 (0.12)  [0.93, 1.30] | 1.14 (0.11)  [0.93, 1.30] |
| Aiming (MT)  - Better Eye | 1.06 (0.14)  [0.77, 1.50] | 0.98 (0.09)  [0.85, 1.13] | 1.07 (0.15)  [0.85, 1.36] |
| Aiming (MT)  - Both Eyes | 1.12 (0.17)  [0.75, 1.45] | 1.09 (0.14)  [0.89, 1.31] | 1.13 (0.15)  [0.94, 1.36] |

***S2 Table.* Grouped means (sd) [min,max] for Aiming data.** Showing participants with normal VA (Normal); participants with high visual acuity (VA > 0.2 logMAR in with eye; High); Those with a large interocular difference (a difference in VA > 0.2 logMAR between their eyes; Different). MT – Movement Time (s).
