# Supplementary material for: Exploring the effects of degraded vision on sensorimotor performance
Source: PLoS One. 2021 Nov 8;16(11):e0258678. doi: 10.1371/journal.pone.0258678 (PMC8575268; doi:10.1371/journal.pone.0258678)
Supplement: S3 Table — Showing participants with normal VA (Normal); participants with high visual acuity (VA > 0.2 logMAR in with eye; High); Those with a large interocular difference (a difference in VA > 0.2 logMAR between their eyes; Different). pPA–Penalised Path Accuracy (mm). (DOCX) [file pone.0258678.s003.docx]

| Mean (sd)  [min, max] | Normal  (n = 55) | High  (VA > 0.2 either eye) (n = 11) | Different (VA diff > 0.2 between eyes) (n = 11) |
| --- | --- | --- | --- |
| Steering (pPA)  - Worse Eye | 0.89 (0.28)  [0.57, 2.27] | 0.85 (0.21)  [0.61, 1.28] | 0.91 (0.20)  [0.61, 1.28] |
| Steering (pPA)  - Better Eye | 0.84 (0.13)  [0.60, 1.31] | 0.80 (0.14)  [0.64, 1.03] | 0.83 (0.12)  [0.64, 1.03] |
| Steering (pPA)  - Both Eyes | 0.81 (0.14)  [0.56, 1.23] | 0.72 (0.08)  [0.59, 0.84] | 0.80 (0.11)  [0.65, 0.98] |

***S3 Table.* Grouped means (sd) [min,max] for Tracking data.** Showing participants with normal VA (Normal); participants with high visual acuity (VA > 0.2 logMAR in with eye; High); Those with a large interocular difference (a difference in VA > 0.2 logMAR between their eyes; Different). pPA – Penalised Path Accuracy (mm).
