# Supplementary material for: Exploring the effects of degraded vision on sensorimotor performance
Source: PLoS One. 2021 Nov 8;16(11):e0258678. doi: 10.1371/journal.pone.0258678 (PMC8575268; doi:10.1371/journal.pone.0258678)
Supplement: Attachment — Submitted filename: plosReviewerResponse.docx [file pone.0258678.s004.docx]

Dear Editor and Reviewers

We would like to thank you for such useful feedback and comments on our manuscript. We hope we have addressed all concerns below and the revised manuscript. All changes to the manuscript have been made using red font and details of these can be found below, where we respond to each comment in *red italics*.

**Reviewer 1**

This study examined the effect of impaired vision on the performance of several motor tasks: aiming, pegboard, and water pouring in Exp 1, and aiming, tracking and steering in Exp 2. The goal in experiment 1 was to assess the effects of degraded binocular vision while experiment 2 compared performance during binocular and monocular viewing. This is an interesting study with a large cohort of young participants which provides some insight into how short term disruption in vision affects motor performance. The study is well motivated, the design and analysis are appropriate; however, I have a few comments for the authors to consider to improve their paper.

*We thank the reviewer for their interest and comments and hope we have responded to their concerns below.*

Experiment 1

1. Provide more details about the method used to select the Bangerter filters. Did all participants wear the same strength of the filter such that VA was ~0.3 logMAR? If there was a range of filters used, provide the details (mean, std dev, range). This could be summarized in a Table. In case the same filter was used, its effect on VA, CS and stereo could also be summarized in a Table (ie report the mean, std dev and range) or present the distributions using a boxplot rather than just the mean and std dev which is currently plotted in Fig 2.

*Thank you for this comment, I now see that this section was lacking in clarity and I have attempted to make this point more explicit by stating that all participants were fitted with 0.3 logMAR Bangerter filters (page 9, line 180 - 182) but that the effects of these on the visual acuity would vary depending on their level of vision in the first place. I have also replaced figure 2 (now Fig 3, page 17) with a boxplot (as you suggest) to facilitate easier interpretation, so VA, CS and stereopsis are now displayed in a boxplot instead. Thank you for this suggestion.*

2. In Fig 2 mean stereoacuity in no impairment condition is ~100 arc sec which seems too high in a cohort of visually healthy young individuals. I would expect 40 arc sec based on the clinical test, please explain

*We think the mean was being dragged up by one or two participants with very poor stereopsis scores. The new figure 3 (based on your suggestion in the point above re switching to boxplots, which we have done for all visual measures, not just VA) shows the median value which is closer to the 40 arc sec you expect.*

3. Was there only one trial per visual condition for the water pouring and pegboard tasks? Were participants allowed to practice the task before data collection?

*Again, this is a good point, that was in need of clarification. Participants were not given an opportunity to practice, but the tasks were straightforward, and participants had the opportunity to ask for clarification if they weren’t sure what was required of them. Nonetheless, the potential effect of lack of practice was mitigated by counterbalancing the order of the visual conditions between participants. This has been explained in the manuscript on page 12, line 272 - 276.*

4. Was performance on the water pouring task measured with a stopwatch to the nearest tenth or hundredth of a second?

*Temporal aspects of performance on the water-pouring task were measured with a stopwatch to the nearest 1/100th of a second. This has now been clarified on page 10, line 223.*

5. Please provide a more detailed description of the aiming task: what were the instructions: speed, accuracy or both? what was the distance between the dots? The size of the dots? Duration of the interstimulus interval? Sampling rate? The authors mentioned ‘several kinematic measures’ on Line 238 but only one measure is defined in this section, that is ‘movement time’ – based on the definition provided, it sounds like the measure is ‘response time’ rather than ‘movement time’, please clarify. Was accuracy also quantified?

*Apologies for omitting these details. We had initially thought about directing people to the Flatters’ paper that outlines these tasks, but appreciate it’s much easier for the reader to find them here. Please see page 11 for the additional details regarding the aiming tasks. Raw position of the stylus and target are recorded so we can report on and analyse different kinematic measures, but we chose to only present MT here for reasons of brevity. Accuracy wasn’t quantified. Thank you for pointing out our mistake with the definition of movement time. What we had written was indeed a definition of response time, but what we actually present is the time from leaving one target to arriving at the next (movement time).*

6. Paragraph on lines 369-376. This paragraph implies that reaching performance is significantly impaired by induced blur, however, deficits in reaching have not been reported in ref 55, that study found differences in secondary saccades. Also, if water pouring performance is affected it’s probably due to difficulty in seeing the marking on the beaker that needs to be filled with water rather than increased uncertainty in the position of the hand (proprioception provides input about the position and movement of the hand)

Apologies for this oversight, in retrospect this argument is somewhat muddled and your suggestion that the reduction in performance is due to difficulties with seeing the small marks on the beaker/jug seems far more elegant/likely. We have therefore decided to remove the argument based on the work of Niechwiej-Szwedo et al., and have replaced this with your suggestion (page 23, line 393 – 396).

Experiment 2

7. The authors should provide a more compelling case for selecting a binocular vs monocular viewing rather than the three viewing conditions studied in experiment 1. Given the findings in experiment 1, why were the same conditions not studied for the tracking and steering tasks? The logical link between experiment 1 and 2 is missing.

*We are grateful to the reviewer for highlighting our ‘curse of knowledge’ and not making the logic more explicit. Experiment 1 was deliberately designed to mimic the extant literature on this topic. This is why we adopted clinical measures (peg board and water pouring) whilst extending this traditional approach by including sophisticated kinematic analyses. Replication is, of course, the hallmark of good science so we wanted to first confirm an impact of viewing condition on sensorimotor performance as reported widely within the literature and establish whether the planar task also revealed difference as a function of viewing condition. The exploratory study then allowed us to logically progress to the hypothesis driven second experiment. The design of the second experiment was based on the results from the first experiment (where we observed a difference between no impairment and bilateral impairment viewing conditions – but were unable to interpret the unilateral impairment condition) and the desire to test the specific hypothesis of interest. We have endeavoured to make the logic clearer in the revised manuscript (p.24).*

*Notably, in the water pouring and aiming tasks, there were no statistical differences between the unilateral impairment condition and both the no impairment and the bilateral impairment conditions, but the failure to reject the null hypothesis means that these results are difficult to interpret: The lack of a difference between unilateral impairment and no impairment suggests that the unilateral filter has no impact. In contrast, the lack of a difference between unilateral impairment and bilateral impairment suggests that a unilateral filter creates similar impairment to filters in front of both eyes. If we consider the pegboard task, then the post-hoc test would indicate that the unilateral filter has no impact (i.e. no statistical difference with the no impairment condition but statistically different to the bilateral impairment condition). This result seems counter-intuitive and is, in turn, difficult to interpret with reference to the other two tasks. These results illustrate that exploratory studies have limitations when going beyond the identification of main effects, and highlights the need for hypothesis driven experiments once exploratory studies have identified a main effect, hence the change in approach with different conditions that would allow us to interpret the results more easily.*

There is an extensive literature that compared motor performance on various tasks during binocular and monocular viewing that has not been mentioned at all. Are the tasks studied here unique and provide additional insight about the contribution of binocular vision to feedforward and feedback control?

*The literature is indeed extensive and we tried to provide a reasonably comprehensive review of typical studies in the General Introduction. We have made reference to some additional papers (line 421 – 422, 435-440) in the revised version in the Expt 2 Intro but we note that all of the extant papers are exploratory in nature (which is not a criticism – this is the nature of science) and our paper is novel because it is the first to specifically test a hypothesis about the role of binocular vision in tasks of this type.*

The authors should explain why they expect that ‘monocular viewing creates lags in the online feedback system’ - line 413.

*Apologies for not being clear. We have expanded on this on p.26, line 453-457.*

8. Please provide more details for the tracking stimulus: size of the moving circle, speed, size of the stimulus (ie figure 8 path)

*As with the aiming task apologies for not providing sufficient detail and please find amendments on p.27, line 487-490.*

9. Was there a practice trial for any of the tasks?

*Participants were not given an opportunity to practice, but the tasks were straightforward, and participants had the opportunity to ask for clarification if they weren’t sure what was required of them. Nonetheless, the potential effect of lack of practice was mitigated by counterbalancing the order of the visual conditions between participants. This has been explained in the manuscript on page 29, line 523-526.*

10. I think that presenting the pPA measure is not sufficient, it is very hard to interpret this score (unless the reliability and validity of using this score has been established in previous published work - please provide a reference). Was the path accuracy quantified using RMSE? Please present the spatial and temporal accuracy scores separately to facilitate interpretation.

*Penalised path accuracy has previously been used to demonstrate differences in coordination between groups of differently aged children in a paper previously published in Plos one (see Flatters et al., 2014). We feel that this is the best option for this paper as it has been shown to have discriminatory power as well as incorporating both accuracy and an element of time control (as this was not a ‘do it as quickly as you can’ task). Path accuracy was quantised and recorded as the arithmetic mean of the distance between the stylus position and the ideal path (in mm) at each time point through the trial, not RMSE, we have now explained this on page 28, line 505-507.*

*We can present the data separately, but for reasons of brevity and because we feel it surplus to requirements we chose not to. Participants who failed to stay in the timing box and completed the task really quickly were likely to not be very accurate, likewise those that took too long and lagged behind the timing box would have had more time and consequently could have been more accurate. Penalised path accuracy allows us to take these variations in timing into account.*

11. Why was the left eye assigned as the better eye in cases of equal VA rather than using a test to determine the preferred eye (as was done in exp 1)?

*We tested monocular performance with both eyes in the second experiment so ‘eye preference’ was not the same potential issue as it was in the first experiment (where we only tested monocular viewing with one of the eyes). In the second experiment the only implication of assigning the left eye was the testing order for the eyes – so we adopted an approach that minimised the burden we placed on the participants within the testing regime (which is, of course, an ethical requirement).*

12. In the legend of Fig 7 the authors suggest that ‘fatigue’ contributed to poorer performance on the ‘with guide’ condition at the fastest speed. This task was done first and the whole protocol is <15 min, why would fatigue have such a large effect on this task condition? What is contributing to the fatigue? I doubt it’s muscle fatigue. Perhaps there is an alternative explanation

*Thank you for your comment, I can see where this point lacked clarity. As the task is fairly simple, we feel that mental fatigue/boredom is the most like cause of this reduction in performance, we have updated the manuscript on page 31 line 565-567.*

13. Additional analysis was conducted by excluding participants with reduced VA (n=16) or IOD greater than 0.2 (n=11), rather than excluding the data, consider presenting the means for the different groups in a Table or running an exploratory sub-analysis to compare the performance of the individuals grouped by VA. Or perhaps a regression analysis would provide some insight into the contribution of VA to motor performance during binocular or monocular viewing.

*Thank you for your comments. We feel that the analysis with the participants removed adds value to the manuscripts as it reinforces that the results that we are showing are robust to significant disturbances to normal vision. We have, however, added a table including the descriptive statistics for each group to the supplementary materials to add clarity/transparency to the analysis, as suggested.*

*Having re-visited this analysis it was noticed that the coding of some participants excluded as part of the reduced VA group was incorrect. Whereby, some participants who had VA scores of 0.20 logMAR had also been excluded when the methods stated that participants with VA > 0.2 logMAR in either eye had been excluded. To this end, the analysis was re-run with the correct participants excluded (N = 11). Generally, the pattern of results remained the same, however, for the steering task the difference between both eyes and better eye disappeared, but none of the conclusions have changed (see line 590 – 596, page 33).*

14. Why do aiming and steering tasks require fast online corrections (Line 608)? Is there any evidence in this study supporting this interpretation? Lines 248-250 mention that the last 25 aiming trials have a jumping target, and that these trials were excluded from the analysis. If the authors are interested in fast online corrections, analysis of these trials would actually provide some insight into that question.

*These tasks require fast online corrections because humans are noisy biological creatures (e.g. perceptual, neural, muscle noise) and need to continually use visual feedback to correct their reasons. This is an established fact within the sensorimotor literature and we provide a supporting reference [#66] in the revised manuscript. We chose not to analyse the double step target trials as these trials reflect individual differences in the ability to make motor corrections – which means that any results would be difficult to interpret as such individual differences could interact with the viewing condition. We were specifically testing the hypothesis that binocular vision would confer an advantage in non-perturbed conditions and our analysis was designed with this in mind.*

15. Perhaps the argument starting on Line 620 could be presented more clearly, when performance declined during monocular viewing it’s due to the poor acuity in that the viewing eye rather than the IOD?

*Many thanks for bringing this to our attention. I think we were conflating two separate arguments here such that out primary one got lost. We are referring to the idea that there might not be a difference in performance when the ‘worse’ eye is used compared to when the ‘better’ eye is used because the ‘worse’ eye is not that different to the ‘better’ eye in terms of VA. I think using IOD is probably the confusing aspect as it makes the reader think of the binocular vision condition. We have reworded to try and improve clarity (line 620-622, 627-628, page 35). In so doing we have also removed reference to the Domkin study that doesn’t actually support our main point, and also deleted quite a lot of text that was repetitive in terms of our findings. Hopefully this improves clarity and readability.*

16. Paragraph starting on line 640 acknowledges the limitations of the current study and suggest that kinematics could provide more insight into motor performance which may provide some advantages for clinical assessments. In general, I agree with the authors, but it’s important to also acknowledge that the subset of tasks used in this study is quite limited, only planar movements on a tablet, most of daily activities require interacting with objects in a 3D world. Also, there are many other ‘sophisticated kinematic measures’ – it might be worthwhile to provide a brief discussion of why the selected measures were chosen.

*Thank you for this point. We agree and have acknowledged this in the general discussion (p. 37, line 682-688)*

**Minor comments**

- Please proof read and correct grammatical and syntax errors.

*We hope we have edited any syntax and grammar issues.*

- Line 227: clarify what ‘incorrectly placed pins’ refers to, were these pins dropped? Or placed in the wrong hole?

*No participants placed pins in the incorrect hole, so this refers to dropped pins. We have clarified this in the MS (p. 11, line 232-234)*

Figures should be numbered in the order of appearance, in the current manuscript Fig 6 is referred to first on Line 231.

*We have re-ordered the figures (page 11, line 236 and throughout).*

Figure 1: correct the order of steps: currently Step 5 comes after Step 1

*Thank you for pointing out these errors, they have now been amended.*

**Reviewer #2:** The authors present a well written manuscript examining the effects 
of degraded monocular on a series of visual tasks. The demonstrated several 
effects comparing monocular vs. binocular viewing conditions and best eye vs. worst eye, showing that degraded monocular vision usually impairs performance. 
 
The manuscript is well presented, my primary request would be for the authors 
to provide motivation in the introduction for studying young people with normal vision as the mansuscript seems to initially be focused on how this may impact visual impairments in older individuals might impact quality of life.

*We are pleased you like the presentation of the MS. We have elaborated on the motivation as per your suggestion (Intro p.4 line 82).*

The results show that even in this healthy population deficits are often apparent so it stands to reason these may generalise to an older population with actual visual problems (although as mentioned in the discussion this may be overcome to some degree with training), further it would be interesting to have same age comparisons with people who are stereo blind (for another study but worth mentioning in dicussion) 

*Thank you for this suggestion. We have included more discussion on future research plans on p.37, line 675-679 and directly addressed the idea of investigating the effects in people with visual deficits including those who are stereoblind, and older adults specifically.*

L65 what is the functional impact of "a" unilateral visual deficit?

L144 capitalize name "Purdue Pegboard Task"

L197 delete repeated "consisted of" 
L210 do you mean participants who correctly identified just 1 circle and 
not "the 1 correct"?

*Thank you for picking up on these typos. We have fixed them all*

Could you add asterisks highlighting the significant comparisons in the charts 
presented in Figures 2-5, 8

*These have now been added and bring additional clarity to the plots*

L412 what about rivalry? Perhaps performance is made worse due to this. 
I am not sure what you mean by creating lag can you be more specific. 
For example, rivalry might cause uncertainty about stimulus location which might slow movements. Is there another mechanism you are thinking of? Perhaps that binocular summation generates a stronger neural signal that might be processed faster - can you cite prior research backing this idea? 

*We don’t believe that binocular rivalry can explain these results as dissimilar monocular stimuli were not presented – rather one eye was occluded. We agree with the reviewer’s suggestion about binocular summation and we apologise that we didn’t make this clearer within the original manuscript. The online feedback system needs to detect when the end-point effector (the stylus tip in our experiments) has moved away from the desired path. This means the visual gap between the position of the stylus and the desired path must reach the threshold for detection. The increased sensitivity associated with binocular vision means that the detection of a disparity between actual and desired position will be faster under binocular vision and this will, in turn, allow a more rapid correction of the movement trajectory. Conversely, there will be a relative delay (lag) in response under monocular viewing conditions. We now make this argument explicit within the revised manuscript and include a citation (p.26, #65, line 448-453).*

L431 - Participants weren't compensated? Were they given course credit please clarify?

*In Experiment 2 participants were recruited through opportunity sampling and not compensated financially or through course credit for their time despite this being the case in experiment 1.*
