# Supplementary material for: Exploring the effects of degraded vision on sensorimotor performance
Source: PLoS One. 2021 Nov 8;16(11):e0258678. doi: 10.1371/journal.pone.0258678 (PMC8575268; doi:10.1371/journal.pone.0258678)
Supplement: Attachment — Submitted filename: plosReviewerResponse2.docx [file pone.0258678.s005.docx]

Dear Editor and Reviewers

We would like to thank you for such useful feedback and comments on our manuscript. We hope we have addressed all concerns below and the revised manuscript. All changes to the manuscript have been made using red font and details of these can be found below, where we respond to each comment in *red italics*.

**Reviewer 2**

Also, as far as I could see the manuscript does not include a reference or link to where the open data can be accessed - I may be in error but please make sure this is present to meet the PlosOne open data policy

*Thanks. We have added the link in the paper (see line 303-305, page 16 and line 562-563, page 31).*

L239 - Please list average estimated visual angle and distance of viewer to the tablet screen.

*We have added this detail in text (see line 242, page 11).*

L235 - PLease clairfy in text and caption that participants only do the aiming task
in Exp 1 and that the other tasks are relevant to Exp 2

*We have added this detail in the text (see line 249-250, page 11) and in the figure caption (see line 274-276, page 14).*

L272 - please clarify - I think you mean to say:
All baseline tests for performance with full vision baseline were recorded first for each participant with no time allotted for practice as the conditions are easily learned. After baseline recordings, conditions with simulated visual impairments were counterbalanced.

*Conditions were fully counterbalanced, including the no impairment/full vision condition e.g. not everyone did the full vision/no impairment condition first. Apologies that this was not clear. We have altered the text accordingly (see line 278, 280-281, page 14).*

L335 Is there a need to present the interaction between speed & accuracy? The findings make sense but I'm not sure what it illustrates that aren't already evident in Figure 4A & 4B? I'm fine either way but if the authors prefer to keep can you add a sentence or two highlighting why this is useful and what it adds beyond time and accuracy alone.

*Thank you. We would prefer to keep it in due to the emerging marginal difference between no impairment and unilateral impairment, so have added a statement about why it’s useful on line 225-227, page 10.*

L389 The accuracy x time interaction is mentioned again and it is mentioned as novel but with no further description of its utility - Please to add some justification on why it is useful beyond considering time and accuracy alone, Else please omit as described above.

*In addition to the text on line 225-227, page 10 we have also addressed this on line 404-405, page 24.*

L473 - Please state the average and std.dev. of the difference between worst and best eye and the sd?

*We have added this information on line 481-482, page 28.*
